# Water as the often neglected medium at the interface between materials and biology

**DOI:** 10.1038/s41467-022-31889-x

**Published:** 2022-07-21

**Authors:** B. L. Dargaville, D. W. Hutmacher

**Affiliations:** Max Planck Queensland Centre on the Materials Science for Extracellular Matrices, Queensland University of Technology, Brisbane, QLD 4059 Australia

**Keywords:** Implants, Biomaterials, Small molecules

## Abstract

Despite its apparent simplicity, water behaves in a complex manner and is fundamental in controlling many physical, chemical and biological processes. The molecular mechanisms underlying interaction of water with materials, particularly polymer networks such as hydrogels, have received much attention in the research community. Despite this, a large gulf still exists in applying what is known to rationalize how the molecular organization of water on and within these materials impacts biological processes. In this perspective, we outline the importance of water in biomaterials science as a whole and give indications for future research directions towards emergence of a complete picture of water, materials and biology.

## Abstract (web summary)

Key to life, water is an often overlooked or over-simplified factor in materials and biology. In this perspective the importance of water in biomaterials is discussed as well as the need to more carefully consider water’s role in every aspect of a biomaterial’s function.

## An exceptional molecule

Water is central to all life. Cells, whole organisms and indeed entire ecosystems are fundamentally and completely dependent upon the presence of water. Water is the most abundant substance on earth, making up around 70% of the earth’s surface and 65–90% of the mass of living organisms^1^. Hence, the importance of water in biological processes has been receiving attention in the scientific community for well over a century^2,3^.

Water plays an important role in all vital processes of living organisms. All facets of the structure and function of both cells and the extracellular matrix (ECM) are centered around the physical and chemical properties of water. Broad biological functions of water include its action as a transport medium for nutrients and waste products, a medium for chemical reactions, cellular osmoregulation and maintenance of cell turgidity, body temperature regulation, lubrication, pH regulation and the formation of pH buffers.

Water is a complex, structured liquid. It dissolves most biologically important molecules (the notable exceptions being lipids and some amino acids). On the other hand, it is much more than just a passive solvent. Water molecules participate actively as a nucleophile and/or proton donor or acceptor in many chemical reactions in living organisms, such as photosynthesis, cellular respiration, condensation reactions, and hydrolysis of both endogenous and foreign compounds. In addition, over the last several decades it has become apparent that water also plays an active role in many other aspects of the human body and its interaction with foreign substances and surfaces^4^. Much of our understanding of the role of water in biological systems stems from studies of protein and DNA in aqueous solution. Protein-ligand binding, as occurs in the immune response for example, has been suggested to be in part determined by the energetics and dynamics of water^5^. Water—at times individual molecules— facilitates enzyme catalysis and water molecules strongly bound to biomolecules impart thermodynamic stabilization to the latter^5^.

Central to the chemical and physical behavior of water is its nature as a polar molecule. The hydrogen and oxygen atoms have vastly different electronegativities. Thus, the oxygen atom carries a partial negative charge due to its greater attraction for the shared electrons of the H-O covalent bond. Consequently, the two hydrogen atoms carry a partial positive charge. The formation of this dipole results in electrostatic attraction between H and O atoms of adjacent water molecules, generating a type of secondary bonding called hydrogen bonding. Hydrogen bonds are weaker than covalent bonds. The hydrogen-oxygen bond dissociation energies are 21 and 464 kJmol^−1^ for hydrogen bonds and covalent bonds, respectively^6^. Therefore, within the temperature range for which water is a liquid, hydrogen bonds are able to break and re-form in a continuous dynamic fashion. The lifespan of a hydrogen bond in liquid water is in the range of tens of femtoseconds to picoseconds^7^. Although individual hydrogen bonds are weak, collectively they result in the high cohesive forces of water as a substance^8,9^.

The polar nature of water enables crucial cellular functions such as cell membrane formation, support of the three-dimensional shape of the DNA double helix, and it has an important role in the tertiary structure of proteins—specifically, water enables hydrophobic interactions, which are crucial to protein folding and aggregation^10^. Water is a polar, protic solvent and amphoteric reagent, and has the ability to ionize both itself and other molecules. Due to its high heat capacity, water protects against the effects of temperature fluctuation.

There is a vast amount of literature on the behavior of water. The picture is, as yet, far from complete and research in this area is highly active. Entire journal issues have been devoted to the topic of water and its properties^11^. Water is unique among all chemical substances in that it displays many anomalous and unexpected behavior parameters. For example, water has an unusually high boiling point for a substance composed of such small molecules; water displays a decreased viscosity when under pressure; it shows a maximum density at 4 °C and ice has a lower density than liquid water. Other thermodynamic parameters, such as specific heat (*C*_*P*_), thermal expansion coefficient (α_P_), and compressibility (k_T_), all show anomalous behavior^12^. Much of the unusual behavior of water is linked to hydrogen bonding. For example, the high heat capacity and heat of vaporization are due to the large energy input required to break up the hydrogen-bonded network to allow greater molecular movement.

Pettersson et al stated that one of the central questions to the understanding of water is ‘What are the structure and dynamics of the hydrogen bonding network that give rise to its unique properties?’^11^ The introduction of ions and interfaces further complicates the unique properties of water and such scenarios are less well understood than those involving bulk water^11^.

The interaction of water with macromolecules is important on a number of levels, ranging from water associated with both ECM and cellular components such as proteins^13,14^ to water interacting with drugs, medical devices and implants^15–17^.

The concept of ‘biological water’ has gained prominence in the recent literature. It has been variously defined as any water surrounding a biomolecule; a shell of functional water surrounding a biomolecule; to the notion of cellular water as a distinctive species able to itself perform biological functions^18^. In any event, there is no dispute that a layer of water exists around biomolecules (and other macromolecules), whose properties differ considerably to those of bulk water. In this Perspective Article, we focus on the interaction of water with biomaterials, and more specifically hydrogels, rather than the role of water in the function of endogenous biomolecules.

## Hydrogels as the key to understanding the water interface

Water’s exceptional behavior, coupled with its importance in biological systems, has prompted generation of a large body of work with respect to investigation of its properties in the context of biomaterials. Hydrogels are among the most widely used biomaterials for healthcare applications. In addition, hydrogels have been used for applications in other fields such as agriculture, food technology and hygiene. However, biomedical applications represent by far the largest sector, with hydrogels being used widely in areas including pharmaceuticals^19^, diagnostics, tissue engineering and regenerative medicine^20^, drug delivery^21,22^, wound dressing, biofiltration, and biosensors^23,24^.

A hydrogel is a three-dimensional network of crosslinked hydrophilic polymer chains. The network can swell and hold a large volume of water and has the integrity of a semi-solid material. By definition, a hydrogel contains at least 10 % of its weight or volume as water but may absorb many times its weight in water. The high water content results in mechanical properties similar to natural biological tissue. A distinguishing property of hydrogels is their response to external physical and chemical stimuli, such as temperature, pressure, pH, solvent composition, and the presence of ions and other dissolved species. In this respect and many others, hydrogels have the potential to closely simulate natural biological environments.

Hydrogels can be classified according to their origin (natural, semi-synthetic or synthetic), their ionic charge (cationic, anionic or neutral), or the type of crosslinking involved (covalent, physical, ionic, and others).

Natural hydrogels are derived from polymers such as collagen, gelatin, agarose, alginate, fibrin, chitosan, and hyaluronic acid^25^. Natural hydrogels have been extensively used in numerous biomedical applications, specifically drug delivery and tissue engineering and regenerative medicine research, due to their inherent biocompatibility, biodegradation and bioactivity, such as promoting cell growth^26^. Synthetic hydrogels are based on polymers or copolymers, which include poly(ethylene glycol) (PEG), poly(vinyl alcohol) (PVA), synthetic polypeptides, poly (N-vinyl-2-pyrrolidinone) (PVP), and poly (2-hydroxyethyl methacrylate) (PHEMA). Synthetic hydrogels are, in general, less biocompatible and biofunctional than natural hydrogels; however, they have better mechanical properties and can be easily tailored to different requirements by varying the synthesis parameters.

**Fig. 1:**
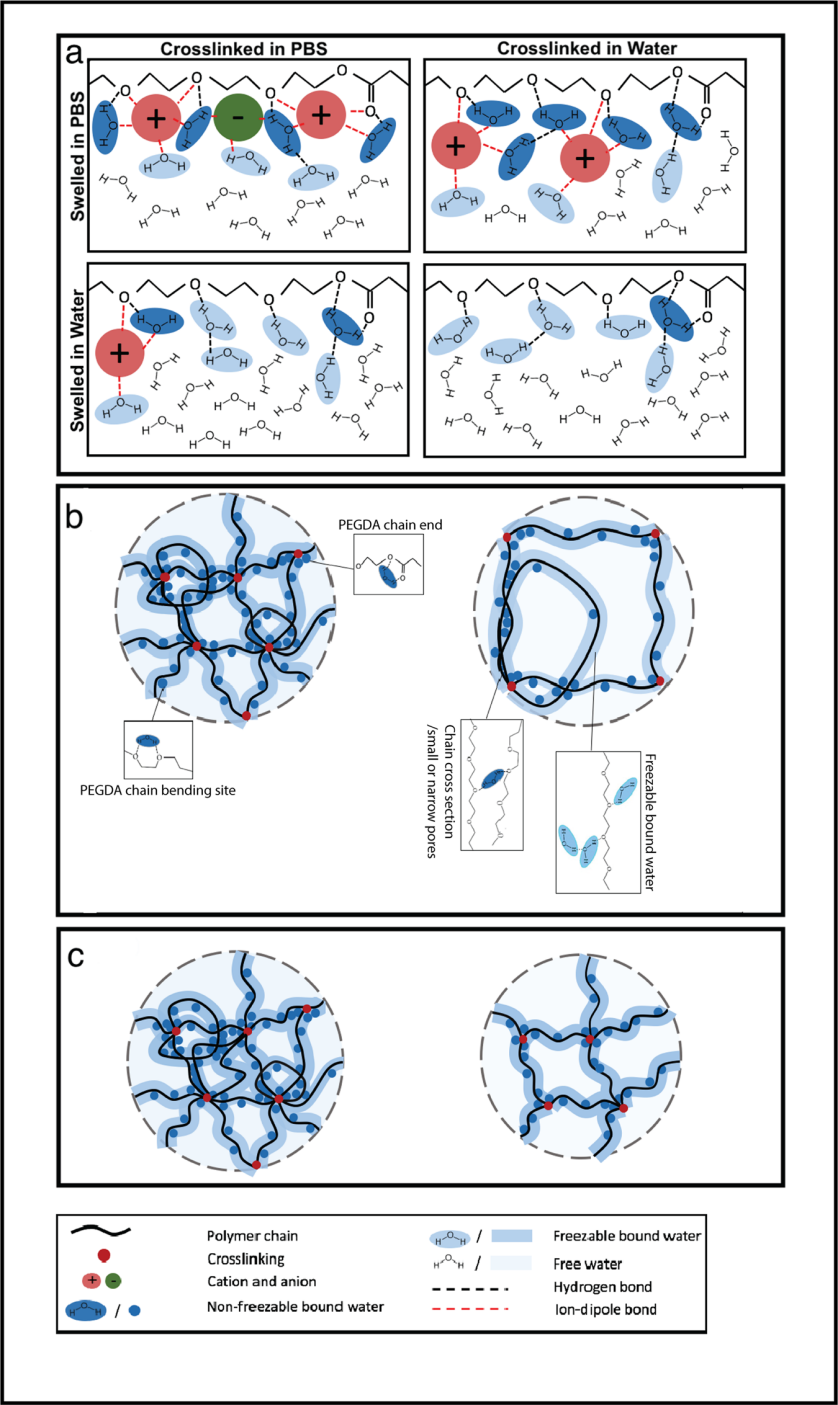
Graphic illustration of water content in PEGDA hydrogels. **a** Water content under different swelling conditions. **b** Comparison of water content in hydrogels with different PEGDA molecular weight. **c** Comparison of water content in hydrogels with different PEGDA weight fraction. (Figure reproduced from Yang et al, *Polymers*, 2021, 13 (6), 845, 10.3390/polym13060845).

The overall structure of hydrogels is determined by the type of polymer matrix, degree of crosslinking, porosity and pore structure. However, the central theme of all hydrogels is water. Many of the physical properties of hydrogels are closely related to the water content and organization of water both within the gel and at the gel surface (Fig. 1). This organization in turn is dependent upon many factors, both internal (related to the gel composition itself) and external (related to the composition of the surrounding environment)^27^.

In the 1970s, 80s and 90s a significant amount of work was done on characterization of water states in hydrogels, and a discussion of this body of work appears below. However, with increasing sophistication of hydrogel systems, both natural and synthetic, developed for biomedical use, such fundamental characterization has been largely neglected in recent years.

Many of the early studies were aimed at identifying the presence of different types of water present in polymer systems, initially in natural systems such as proteins and polypeptides, then also synthetic gels such as those composed of PVP and methacrylate polymers. Various designations have been used for the observed water states, including hydration water, associated water, bound versus free water, fast versus slow water, and freezable versus nonfreezable water. One of the first observations was that water within polymers does not display the usual sharp, first-order thermodynamic phase transitions seen for bulk water^28^. Thermodynamic measurements revealed the step-wise nature of the hydration process^29^. A hierarchy was established for the strength of water interactions within proteins: water-ion > water-water = water-polar group > water-hydrophobic group^30^. Clear evidence was compiled for the presence of hydrogen bonding interactions between water and polymer chains in synthetic polymers^31–33^. It was recognized that the behavior of water is sensitive to many different factors, and that different techniques, such as differential scanning calorimetry (DSC) and nuclear magnetic resonance (NMR) reveal different aspects of the water phenomena. For example, concentration, temperature, plasticization, polymer mobility and conformation, hysteresis effects, crosslinking and the presence of extra components such as salts, all complicate data interpretation^28^.

The cumulative effort of this era resulted in the ‘three-state’ model of water in hydrogels, wherein water exists in ‘bound’, ‘intermediate’ and ‘free’ states^28,34,35^. The bound water forms the primary hydration shell around the hydrophilic polymer chains and does not show freezing/melting behavior. Intermediate water forms the secondary hydration shell and exhibits ice melting below 0 °C due to moderately strong interactions with the polymer chains. Free water does not interact with the network structure and behaves very similarly to bulk water, freezing and melting at around 0 °C. The relative content of the three water states depends on many interacting factors, including overall water content. Although only tacitly acknowledged at the time, this creates a fascinating and complex array of possibilities in terms of implications for the biological response to these systems.

Peppas et al carried out work on the thermodynamic interactions of copolymer hydrogels with biological fluids utilizing the Florey–Huggins thermodynamic theory, for the purpose of the evaluation of biomaterials for different applications^36,37^. They developed a new method for determining the Florey interaction parameter, χ, for hydrophilic copolymers in contact with water. The work of Peppas, as well as others, allowed prediction of swelling characteristics and solute diffusion for hydrogels designed for biomedical applications, having important implications for protein adsorption, mechanical properties, refractive index, and drug diffusion^38^.

Knowledge of the interaction with water is important from both a biological and a material science perspective: polymer properties depend heavily on the degree and nature of water absorption; while cellular interactions depend both directly on water arrangement at the molecular level and indirectly on the properties of the hydrated polymer.

From a material science point of view, the sorption of water is important for the design of biomaterial properties, understanding plasticization phenomena, and understanding the interaction of polymers with other small molecules^39^. The study of water states sheds light on the pore structure (macroporous, microporous and nanoporous) of hydrogels since water exists in different states in different types of pores and voids^27^.

Over the last decade researchers have synthesized new hydrogels with the emphasis on applications and advanced systems containing cells and other active components for therapeutic use^40–42^. The more fundamental research into molecular structure, water content and structure, and how this relates to functionality has, for the most part, lately been forgotten. Many hydrogels, and biomaterials in general, are lacking the level of function necessary for successful integration into biological systems, and consequently their performance is sub-optimal^43,44^. In order to bridge the gap between the emergence of ‘new’ hydrogel systems and successful extension of these to application, a systematic approach is required for the fundamental characterization of the macromolecular interactions in an aqueous environment.

## Probing the water phenomena

In order to research water in hydrogels by experimentation, it is a condition sine qua non to build up a fundamental knowledge of the methods involved and their theoretical interpretation, thus assisting in the understanding of the structure and dynamics of bound, intermediate and free water. The study of dynamic processes such as hydrogen bonding and water states necessitates the use of various complimentary experimental techniques. Different methods give varying perspectives on the ‘water state’ phenomenon, since each gives information at different temperatures, different spatial scales and timescales. For example, DSC shows the presence of distinct states of water at low temperatures (in the vicinity of 0 °C) whereas the concept of different states is less applicable at temperatures above ambient (273 K). However, Fourier transform infrared spectroscopy (FTIR) and thermogravimetric analysis (TGA) can give information on water states above 273 K^45^. Another example of this is that NMR and thermally stimulated depolarization current (TSDC) allow study over different temperature ranges (200–280 and 90–270 K, respectively). There has been considerable debate on how to correlate the results from the different techniques^10^.

Methods, such as DSC and NMR, can be used in parallel to gain a deeper insight into hydration and dehydration phenomena of hydrogels. Such combining of techniques allows for a more comprehensive analysis. For example, the parallel use of DSC and X-ray diffraction (XRD) has been used to monitor the growth of ice crystals during the cold crystallization process of intermediate water in the heating cycle of hydrated biomaterials^46^.

Table 1 outlines the experimental techniques for studying water in hydrogels that have advanced the knowledge base, along with some of the materials to which the techniques have been applied. One of the most utilized methods is DSC, in its various forms. It allows quantification of bound, intermediate and free water within gel systems and represents a good starting point for acquiring an overall picture of water behavior in polymeric systems.

**Table 1:** Some of the techniques available for studying the state of water in hydrogels.

Technique	Information obtainable	Advantages	Disadvantages	Hydrogels studied	References
DSC	Detect and quantify bound, intermediate and free water from thermal transitions and isothermal crystallization; number of water molecules per polymer repeat unit.	Gives a good macro-level view of hydrated systems; can differentiate different degrees of binding (ie. strongly bound vs intermediately bound).	Insensitive to behavior in certain temp. ranges; may not be relevant to hydrogels at ambient temp.; sensitive to scan rate; unable to provide detailed structural info; insensitive to water at very low concentrations.	PVP, PHEMA, PMAA PEG, Polyamines, Chitosan-PVA, PMEA	^28,61–65^
^1^H and ^2^H NMR (T_1_ and T_2_ relaxation measurements)	Detect and quantify bound, intermediate and free water; number of water molecules per polymer repeat unit; Temp. range 200–280 K; microsecond-millisecond timescale.	Can probe a single molecular layer of bound water; is more sensitive than DSC; allows probing of dynamic behavior;	Cannot detect different populations of water if exchange is fast on NMR timescale, in which case only a weighted-average population is observed.	PVP, PHEMA, PMEA Chitosan, Cellulose ethers	^28,35,58,61,66–69^
^1^H pulse field gradient (PFG) diffusion NMR	Allows estimation of bound water fraction from the immobile phase (D = 0).	Gives molecular level information; simple interpretation; can be reliably applied to more complex swelling media eg. cell culture media.	Cannot detect different populations of water if exchange is fast on NMR timescale, in which case only a weighted-average population is observed; doesn’t go beyond a two-site exchange model (ie. bound vs unbound).	PNIPAAm, PHEMA, PHEMA/GMA, PHEMA/PVP, PVP/PMMA, PHEMA/PMA, P(HEMA-co-DHPMA)	^42,70,71^
^13^C NMR	Info. on gel structure and dynamics.	May show how polymer is affected by water (but not vice versa).	^13^C can only provide info. on hydrated polymer structure and not on water itself.	PMEA, PHEMA, PTHFA	^58,72^
Infrared (IR) spectroscopy	Qualitatively identify presence of bound, intermediate and free water; reveals strength of hydrogen bond network.	Allows probing at functional group level for both water and polymer.	Does not give quantitative information; limited to analysis of outer surface for opaque hydrogels; interpretation is complicated by coupling of vibrations between water molecules.	PMMA, PMEA	^73–75^
Raman spectroscopy	Water content based on O-H stretching vibrations; strength and number of H-bonds; reveals strength of hydrogen bond network.	Allows probing at functional group level; direct information about the local structure of water; semi-quantitative.	Interpretation is complicated by coupling of vibrations between water molecules.	PAA, PDMAA, P(MA-co-DMAPMA)	^76–78^
Thermally stimulated depolarization current (TSDC)	Dielectric properties of bound and mobile molecules; dynamics of bound water close to surfaces or macromolecules; temp. range 90–270 K.	Broader temp. range possible than ^1^H NMR and some other techniques. High sensitivity and resolution.	Is an indirect technique; The full potential of this technique has not yet been realized; more work needs to be done on models to fit the data.	PVA, PEG, GAG	^79,80^
Thermogravimetric analysis (TGA)	Detection of ‘fast’ and ‘slow’ evaporating water, allowing quantification of ‘free’ and ‘bound’ water, respectively.	Gives complimentary information to other techniques.	Doesn’t give information on molecular level and needs to be interpreted in combination with other techniques.	P(HEA-co-HEMA)	^81^
Dilatometry	The presence of different states of water from specific volume transition at various temperatures.	A common application of a dilatometer is the measurement of thermal expansion. Thermal expansivity is an important engineering parameter.	Indirect method; only qualitative/semi-quantitative analysis possible; most literature using this technique for hydrogel-water studies is very old.	PHEMA, PDHPMA	^82,83^
Specific conductivity	Activation energy for specific conductivity versus water content shows three distinct zones corresponding to different water states.	Electrical conductivity is highly sensitive to hydrogen content and insensitive to other factors such as other major chemical elements. Consequently, electrical conductivity should be a very good property from which one can infer the water (hydrogen) distribution.	Indirect method; only qualitative/semi-quantitative analysis possible; most literature using this technique for hydrogel-water studies is very old.	PHEMA, PDHPMA	^82,83^
Dielectric relaxation	Correlation of dielectric constant with structure of water phase.	Useful for systems exhibiting multiple phases; the technique has grown in priminence and has been applied in a variety of fields, thus setting the scene for future water-hydrogel studies.	Most literature using this technique for hydrogel-water studies is very old.	PDHPMA, PMEA	^83,84^
X-ray diffraction (XRD)	Length (and hence strength) of H bonds; types of ice crystal formation.	Useful for analysis of short-range structure; coupled XRD-DSC valuable for determining the origin of phase transitions, especially for multi-component materials.	A specialist technique and has limitations. The sample must be single phase and homogeneous. Proper preparation of samples is crucial. If the sample is a non-isometric crystalline structure, indexing its patterns can be complex. Where there are high-angle reflections, peak overlay can occur and complicate interpretation.	PDMAA, PHEMA, PMEA	^46,85,86^
Quasi-elastic neutron scattering (QENS)	States of water via diffusion coefficients of protons, in both water and polymer.	Is unique in probing the motion of atomic nuclei, rather than measuring the response of the electrons to the nuclear dynamics, which is the case for most other spectroscopies. This direct type of interaction makes comparison between neutron scattering and classical molecular dynamics simulations successful and straightforward. It can probe events on pico to nano second timescale (the range of H-bond lifetime).	The question of relative efficiency of QENS is still open. The information required from a QENS experiment is in the time rather than frequency domain, so it is usual to fit the measured QENS signal with model functions that can be transformed to the time domain, to get I(t), but truncation and statistical errors can lead to oscillations in the resulting I(t).	Polysaccharide hydrogels, PEG	^87,88^
Theoretical molecular modeling	Water mobility and diffusion; hydrogen bonding dynamics; hydration energy.	Molecular dynamics simulations have evolved into a mature technique that can be used effectively to understand macromolecular structure-to-function relationships.	Lack of representative standards; much less optimized analysis tools; difficulties in storing and transmitting the huge amount of trajectory data that is generated are still issues that remain to be solved.	PVA, PNiPAM, PVME, Gelatin, PMEA	^27,39,79,89–91^

Although DSC is extremely functional for quantifying the amounts of the three different states of water, it does not detect water below certain scale threshold levels and cannot be used on its own to construct a picture of events on the molecular and functional group scale. Spectroscopic methods are more likely to reveal this kind of information. ^1^H NMR spectroscopy is a more sensitive and much utilized technique. ^1^H, ^2^H and ^17^O T_1_ and T_2_ relaxation measurements are able to give much of the same information as DSC but over a wider temperature range and can probe a single molecular layer of bound water. ^13^C NMR can be used to obtain structural information about the hydrated macromolecules. This can be useful because the network structure and dynamics of the polymer chains may have an important role in regulating the water structure. Yet, direct information about water cannot be obtained from this technique. Infrared spectroscopy allows dynamic probing of the interaction of water with specific functional groups present on the polymer chains but hasn’t thus far allowed quantification of the different types of water present in hydrogels.

Recently, in situ Raman spectroscopy has been employed to identify three distinct types of water, based on differences in their O-H stretching vibrations, in the context of interfacial water involved in electrochemical reactions^9,47^. Distinction was made between 4-coordinated, 2-coordinated and ion-coordinated water molecules. Similar methods could in the future be applied to hydrogel systems to probe in detail the exact bonding arrangements that lead to the different states of water observed in these systems.

Several other less common, and therefore to a lesser extent scientifically validated, techniques have been used by a number of research groups to elaborate further on specific aspects of the behavior of bound, intermediate and free water in hydrogels and representatives of these are presented in Table 1.

## Outlook

**Fig. 2:**
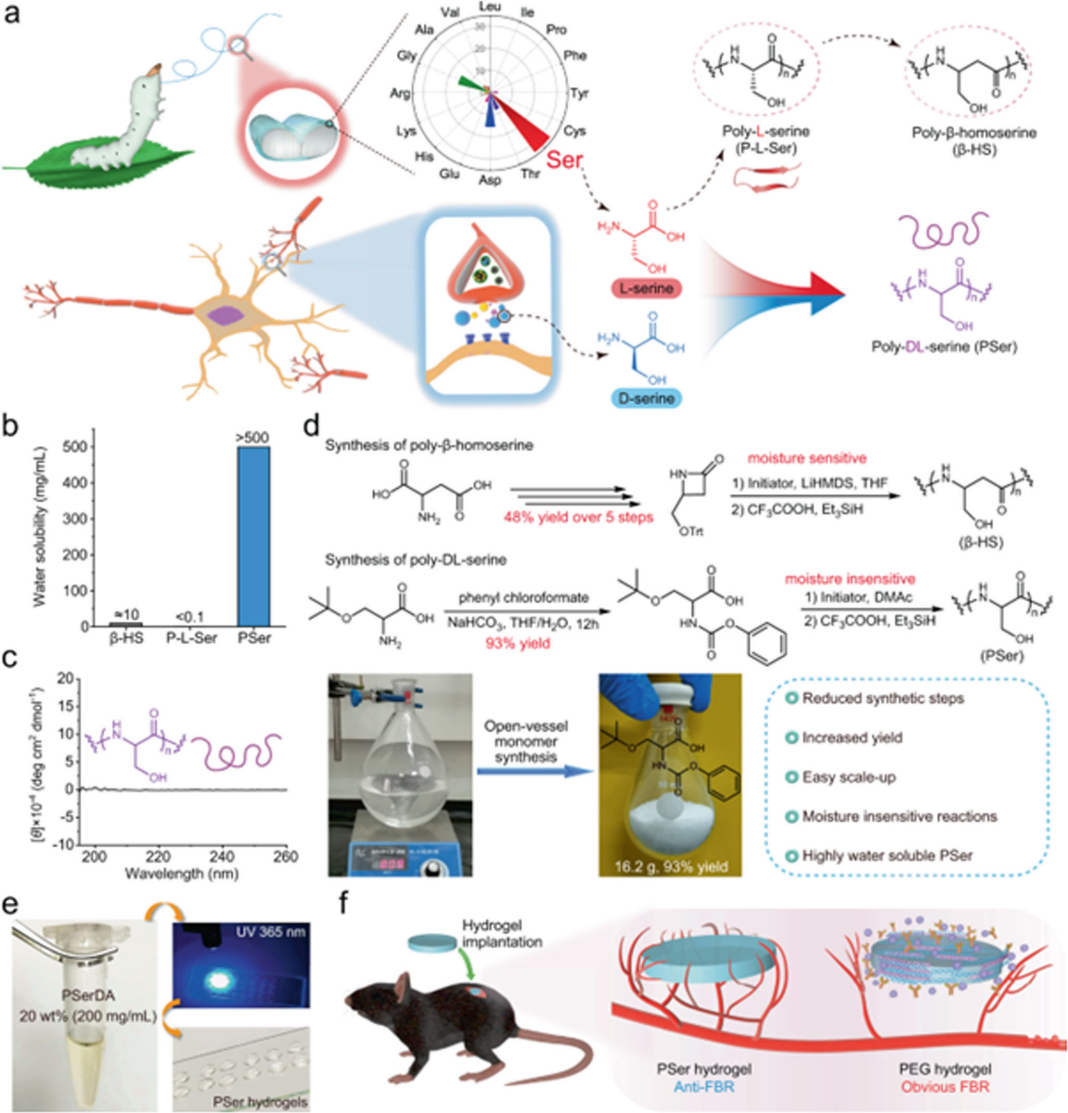
Examples of ‘synthesis to application’ of hydrogels. **a** Design of poly-dl-serine (PSer) from l-serine and d-serine. The high l-serine content in silk sericin and the high level of d-serine in the human body as an important neurotransmitter altogether inspired the design of anti-FBR material PSer. **b** Water solubility of poly-β-homoserine (β-HS) (about 10 mg/mL), poly-l-serine (P-l-Ser) (<0.1 mg/mL due to its β-sheet folding) and PSer (>500 mg/mL). **c** Circular dichroism spectrum of PSer. **d** Synthesis of β-HS and PSer. LiHMDS Lithium hexamethyldisilazide, DMAc dimethylacetamide. **e** Photographs of poly-dl-serine diacrylamide (PSerDA) that was well dissolved at a concentration of 20 wt% and was used to prepare PSer hydrogels by photo-crosslinking in the presence of 0.1% photoinitiator (Irgacure 2959). **f** PSer hydrogels and PEG hydrogels implanted subcutaneously into C57/BL6 mice induced low FBR and obvious FBR respectively (Figure reproduced from Zhang et al. *Nat. Commun.* **12**, 5327 (2021), 10.1038/s41467-021-25581-9).

Hydrogels are of particular interest with regard to the water-biomaterial account because the development of hydrogels for biomedical applications represents one of the most studied areas at the interface of material science, engineering and medicine. The reason for their attractiveness centers around the ability of these water-rich matrices to mimic natural tissues, both physically and functionally (Fig. 2).

The term ‘biocompatible’ is generally used to describe materials which are able to perform a specific biological function while maintaining an appropriate host response. Such materials thus do not give rise to adverse effects when in contact with biological components such as cells, blood and tissues. Specific requirements of biocompatible biomaterials include the ability to resist protein adsorption and cell adhesion, lack of immunogenicity, and being mechanically matched to the host tissue.

The in-depth mechanisms underlying blood and biocompatibility and the overall host response to biomaterials have not been clearly elucidated. However, there have been many attempts in this direction and it appears that the physicochemical properties of hydration water play an important role^48–50^. The blood compatibility of many natural and synthetic polymers has been closely linked to the presence of intermediate water, which appears to prevent direct contact between blood cells, proteins and the material surface^51^. In particular, NMR spectroscopic studies have shown that intermediate water bound to the polymer chains prevents protein adsorption^43^. Other studies have also correlated the presence of hydration water with anti-fouling properties^52,53^. Highly hydrated polymers exhibit resistance to non-specific protein adsorption^54^, and changes in hydration due to copolymerization with hydrophobic monomers or increases in temperature^55,56^, for example, thus lead to a reduction of non-fouling properties.

Attempts have been made to correlate polymer side-chain mobility, subsequent water mobility and biocompatibility from the viewpoint of protein adsorption^57,58^. It was found that the flexibility of both polymer (PMEA>PTHFA>PHEMA) and bound water was directly related to the discovery creating of a marker for biocompatibility, TAT (thrombin-antithrombin III complex)^58^.

As many research groups develop new and more sophisticated hydrogel systems^59,60^ for biomedical applications, with prominence being given to advanced approaches incorporating cells and other active components, the link between translational and fundamental research into the role played by different water states in material end-function is more important than ever.

As described in the preceding sections, the molecular mechanisms underlying the interactions of water molecules with biomaterials, specifically polymer networks such as hydrogels, have been well-studied and although there is much still to be learned, progress has been achieved in understanding these processes. However, a significant gap still exists when it comes to applying this knowledge to rationalize the way in which the molecular organization of water on the surface and within biomaterials affects their in vitro and/or in vivo biocompatibility.

**Fig. 3:**
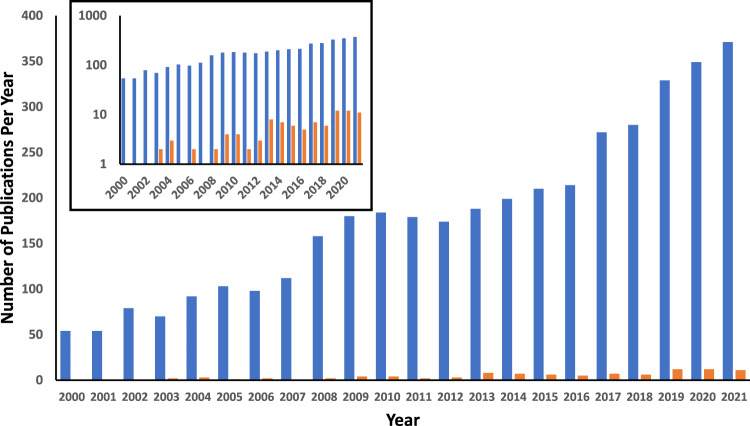
Number of biomedically-relevant journal articles published on hydrogel swelling since 2000 (blue bars) compared to the number of those same papers that mention PBS (orange bars). Inset: The same data presented on a logarithmic scale to enable easier visualization. Source of data: Web of Science. See Supplementary Information for details of the method used to conduct the search.

Biotherapeutic environments are complex and consist not only of tissue, implant and water, but also a multitude of other molecules and species in the cellular and extracellular space. Another layer of the puzzle that warrants consideration concerns the presence of these species (which include metabolites, electrolytes and other osmolytes) and their influence on the interaction of water with hydrogel macromolecules and the subsequent biological response. While it has been anticipated that they will affect the properties of water, exactly how this occurs has been controversial and there is a distinct lack of research considering this problem. Most researchers traditionally frame their experimental models against a pure water setting (Fig. 3). Clearly, the biological relevance of such an approach is limited.

Figure 3 displays the number of papers in the biomedical literature, published since 2000, that have studied hydrogel swelling, compared to the number of those same papers that use PBS as the swelling medium. ‘PBS’ was chosen as a search term in order to represent those studies that have considered more physiologically relevant swelling media than pure water. Although it can be conceded that this data excludes a number of studies which utilize other more complex osmotic and biologically relevant fluids, the number of such studies is very small compared to those that use PBS, and their omission does not alter the overall trend shown. Indeed, a closer perusal of the studies that do *not* involve PBS reveals that most of them do in fact use pure water as the swelling medium.

The rapidly emerging design space of hydrogels with controlled physical, chemical and biological properties represent a great opportunity for future research. We propose that future strategies in this area should involve pairing what is known from the early fundamental research on water in hydrogels with the more recent work on the role of water in biological systems and applying this to the characterization of newly developed hydrogel systems. Detailed studies in this direction will not only enable filling of some of the knowledge gaps that exist hitherto, but will also enable the development of a more systematic approach for hydrogel characterization. Only in this way will a more complete scientific framework of biocompatibility, water and materials emerge. This may indeed require the development of new experimental and theoretical techniques to further probe the dynamics of this topic and answer the many questions that remain regarding the big picture of water in biomaterial science as a whole.

## Supplementary information

Supplementary Information

## References

[1] *A-Level Biology 2015–2021*, https://alevelbiology.co.uk/notes/the-biological-importance-of-water/ (accessed May 2022).

[2] Pantsar T (2022). Decisive role of water and protein dynamics in residence time of p38α MAP kinase inhibitors. Nat. Commun..

[3] Novick KA (2022). Confronting the water potential information gap. Nat. Geosci..

[4] Ball P (2008). Water as an active constituent in cell biology. Chem. Rev..

[5] Bagchi, B. *Water in Biological and Chemical Processes—From Structure and Dynamics to Function* Ch. 6 (Cambridge University Press, 2013).

[6] Young, P. R. *Introductory Chemistry Online*, https://chem.libretexts.org/@go/page/79577 (accessed May 2022) (2020).

[7] Martiniano HFMC, Galamba N (2013). Insights on hydrogen-bond lifetimes in liquid and supercooled water. J. Phys. Chem. B.

[8] Saini L, Nemala SS, Rathi A, Kaushik S, Kalon G (2022). Selective transport of water molecules through interlayer spaces in graphite. Nat. Commun..

[9] Wang Y-H (2021). In situ Raman spectroscopy reveals the structure and dissociation of interfacial water. Nature.

[10] Ball P (2017). Water is an active matrix of life for cell and molecular biology. Proc. Natl Acad. Sci. USA.

[11] Pettersson LGM, Henchman RH, Nilsson A (2016). Water—the most anomolous liquid. Chem. Rev..

[12] Gallo P (2016). Water: a tale of two liquids. Chem. Rev..

[13] Kuntz, I. D. & Kauzmann, W. In *Advances in Protein Chemistry* Vol. 28 (eds Anfinsen, C. B., Edsall, J. T. & Richards, F. M.) 239–345 (Academic Press, 1974). 10.1016/s0065-3233(08)60232-6 4598824

[14] Bellissent-Funel M-C (2016). Water determines the structure and dynamics of proteins. Chem. Rev..

[15] Zhang D (2021). A sandcastle worm-inspired strategy to functionalize wet hydrogels. Nat. Commun..

[16] Hua M (2021). Strong tough hydrogels via the synergy of freeze-casting and salting out. Nature.

[17] Kim S (2021). In situ mechanical reinforcement of polymer hydrogels via metal-coordinated crosslink mineralization. Nat. Commun..

[18] Jungwirth P (2015). Biological water or rather water in biology?. J. Phys. Chem. Lett..

[19] Kashyap N, Kumar N, Kumar M (2005). Hydrogels for pharmaceutical and biomedical applications. Crit. Rev. Ther. Drug Carr. Syst..

[20] Chhibber, T. et al. In *Intelligent Hydrogels in Diagnostics and Therapeutics* (eds Ghosal, A. & Kaushik, A.) Ch. 8, 105–122 (CRC Press, 2020).

[21] Li J, Mooney DJ (2016). Designing hydrogels for controlled drug delivery. Nat. Rev. Mater..

[22] Sharpe LA, Daily AM, Horava SD, Peppas NA (2014). Therapeutic applications of hydrogels in oral drug delivery. Expert Opin. Drug. Deliv..

[23] Culver HR, Sharma I, Wechsler ME, Anslyn EV, Peppas NA (2017). Charged poly(N-isopropylacrylamide) nanogels for use as differential protein receptors in a turbidimetric sensor array. Analyst.

[24] Tavakoli J, Tang Y (2017). Hydrogel based sensors for biomedical applications: an updated review. Polymers.

[25] Chun, H. J., Reis, R. L., Motta, A. & Khang, G. (eds). In *Biomimicked Biomaterials: Advances in Tissue Engineering and Regenerative Medicine* (Springer, 2020).

[26] Spicer CD (2020). Hydrogel scaffolds for tissue engineering: the importance of polymer choice. Polym. Chem..

[27] Gun’ko VM, Savina IN, Mikhalovsky SV (2017). Properties of water bound in hydrogels. Gels.

[28] Quinn FX, Kampff E, Smyth G, McBrierty VJ (1988). Water in hydrogels. 1. A study of water in poly(N-vinyl-2-pyrrolidone/methyl methacrylate) copolymer. Macromolecules.

[29] Rupley, J. A., Yang, P.-H. & Tollin, G. In *Water in Polymers; ACS Symposium Series* Vol. 127 (ed Rowland, S. P.) 111 (American Chemical Society, 1980).

[30] Rowland, S. P. & Kuntz, I. L. In *Water in Polymers; ACS Symposium Series* Vol. 127 (ed Rowland, S. P.) 1 (American Chemical Society, 1980).

[31] Puffr R, Sebenda J (1967). On the structure and properties of polyamides. XXVII. The mechanism of water sorption. J. Polym. Sci. C.

[32] Moy P, Karasz EE (1980). Epoxy-water interactions. Polym. Eng. Sci..

[33] Jelinski LW, Dumais JJ, Stark RE, Ellis TS, Karasz FE (1983). Interactions of epoxy resins with water. A quadruple echo deuterium NMR study. Macromolecules.

[34] Jhon MS, Andrade JD (1973). Water and hydrogels. J. Biomed. Mater. Res..

[35] Sung YK, Gregonis DE, John MS, Andrade JD (1981). Thermal and pulse NMR analysis of water in poly(2-hydroxyethyl methacrylate). J. Appl. Polym. Sci..

[36] Mikos AG, Peppas NA (1988). Florey interaction parameter chi for hydrophilic copolymers with water. Biomaterials.

[37] Peppas NA, Merrill EW (1976). Determination of the interaction parameter, chi, for poly(vinyl alcohol) and water in gels crosslinked from solutions. J. Polym. Sci. Polym. Chem. Ed..

[38] Peppas, N. A. & Lustig, S. R. In *Hydrog*els *in M*edici*ne and Pharmacy: Fundamentals* Vol. I (ed Peppas, N. A.) 57–83 (CRC Press, 1986).

[39] Tamai Y, Tanaka H (1996). Molecular dynamics study of polymer-water interaction in hydrogels. 1. Hydrogen bond structure. Macromolecules.

[40] Tavakoli S, Klar AS (2020). Advanced hydrogels as wound dressings. Biomacromolecules.

[41] Oliva N, Shin M, Burdick JA (2021). Editorial: special issue on advanced biomedical hydrogels. ACS Biomater. Sci. Eng..

[42] Alam TM, Childress KK, Pastoor K, Rice CV (2014). Characterization of free, restricted and entrapped water environments in poly(N-isopropyl acrylamide) hydrogels via 1H HRMAS PFG NMR. J. Polym. Sci. B: Polym. Phys..

[43] Tanaka M, Hayashi T, Morita S (2013). The roles of water molecules at the biointerface of medical polymers. Polym. J..

[44] Zhang D (2021). Bio-inspired poly-DL-serine materials resist the foreign-body response. Nat. Commun..

[45] Gun’ko, V. M. & Turov, V. V. *Nuclear Magnetic Resonance Studies of Interfacial Phenomena* (CRC Press, 2013).

[46] Kishi A, Tanaka M, Mochizuki A (2009). Comparative study on water structures in polyHEMA and polyMEA by XRD-DSC simultaneous measurement. J. Appl. Polym. Sci..

[47] Waegele MM (2021). Water molecules directed to speed up dissociation. Nature.

[48] Nell AE (2009). Understanding biophysicochemical interactions at the nano-bio interface. Nat. Mater..

[49] Stevens MM, George JH (2005). Exploring and engineering the cell surface interface. Science.

[50] Place ES, Evans ND, Stevens MM (2009). Complexity in biomaterials for tissue engineering. Nat. Mater..

[51] Tanaka M, Mochizuki A (2004). Effect of water structure on blood compatibility, thermal analysis of water in poly(meth)acrylate. J. Biomed. Mater. Res..

[52] Morisaku T, Watanabe J, Konno T, Takai M, Ishihara K (2008). Hydration of phosphorylcholine groups attached to highly swollen polymer hydrogels studied by thermal analysis. Polymer.

[53] Chen S, Lingyan L, Zhao C, Zheng J (2010). Surface hydration: principles and applications toward low-fouling/nonfouling biomaterials. Polymer.

[54] Herrwerth S, Eck W, Reinhardt S, Grunze M (2003). Factors that determine the protein resistance of oligoether self-assembled monolayers - internal hydrophilicty, terminal hydrophilicity, and lateral packing density. J. Am. Chem. Soc..

[55] Leckband D, Sheth S, Halperin A (1999). Grafted poly(ethylene oxide) brushes as nonfouling surface coatings. J. Biomater. Sci. Polym. Ed..

[56] Lutz JF, Akdemir Ö, Hoth A (2006). Point by point comparison of two thermosensitive polymers exhibiting a similar LCST: is the age of poly(NIPAM) over?. J. Am. Chem. Soc..

[57] Yamada-Nosaka A, Tanzawa H (1991). 1H-NMR studies on water in methacrylate hydrogels. II. J. Appl. Polym. Sci..

[58] Miwa Y, Ishida H, Tanaka M, Mochizuki A (2010). (2)H-NMR and(13)C-NMR study of the hydration behavior of poly(2-methoxyethyl acrylate), poly(2-hydroxyethyl methacrylate) and poly(tetrahydrofurfuryl acrylate) in relation to their blood compatibility as biomaterials. J. Biomater. Sci. Polym. Ed..

[59] Caprioli M (2021). 3D-printed self-healing hydrogels via digital light procesing. Nat. Commun..

[60] Zhang Y (2021). Differential diffusion driven far-from-equilibrium shape-shifting of hydrogels. Nat. Commun..

[61] Smyth G, Quinn FX, McBrierty VJ (1988). Water in hydrogels. 2. A study of water in poly(hydroxyethyl methacrylate). Macromolecules.

[62] Bouwstra JA, Salomonsdevries MA, Vanmiltenburg JC (1995). The thermal-behavior of water in hydrogels. Thermochim. Acta.

[63] Cursaru B, Stanescu PO, Teodorescu M (2010). The states of water in hydrogels synthesized from diepoxy-terminated poly(ethylene glycol)s and aliphatic polyamines. UPB Sci. Bull. Ser. B.

[64] Wang T, Gunasekaran S (2006). State of water in chitosan—PVA hydrogel. J. Appl. Polym. Sci..

[65] Tanaka M (2000). Cold crystallization of water in hydrated poly(2-methoxyethyl acrylate) (PMEA). Polym. Int..

[66] Capitani D, Crescenzi V, Angelis AAD, Segre AL (2001). Water in hydrogels. An NMR study of water/polymer interactions in weakly cross-linked chitosan networks. Macromolecules.

[67] Baumgartner S, Lahajnar G, Sepe A, Kristl J (2002). Investigation of the state and dynamics of water in hydrogels of cellulose ethers by 1H NMR spectroscopy. AAPS PharmSciTech.

[68] Yamadanosaka A, Ishikiriyama K, Todoki M, Tanzawa H (1990). H-1 NMR studies on water in methacrylate hydrogels. 1. J. Appl. Polym. Sci..

[69] Mochizuki, A. et al. Water structure of poly(2-methoxyethyl acrylate) observed by nuclear magnetic resonance spectroscopy. *J. Biomater. Sci. Polym. Ed.* **31**, 1024–1040 (2020). 10.1080/09205063.2020.1738042 32131705

[70] McConville P, Pope JM (2000). A comparison of water binding and mobility in contact lens hydrogels from NMR measurements of the water self-diffusion coefficient. Polymer.

[71] Barbieri R, Quaglia M, Delfini M, Brosio E (1998). Investigation of water dynamic behaviour in poly (HEMA) and poly (HEMA-co-DHPMA) hydrogels by proton T2 relaxation time and self-diffusion coefficient NMR measurements. Polymer.

[72] Miwa Y, Ishida H, Saito H, Tanaka M, Mochizuki A (2009). Network structures and dynamics of dry and swollen poly(acrylate)s. Characterization of high- and low-frequency motions as revealed by suppressed or recovered intensities (SRI) analysis of C-13 NMR. Polymer.

[73] Gemmei-ide M, Motonaga T, Kitano H (2007). Breaking of the supercooled state of water by a nanocavity with disordered atomic configuration I: freezing behavior of sorbed water into polymethylmethacrylate film as examined by Fourier transform infrared spectroscopy. J. Phys. Chem. B.

[74] Gemmei-Ide M, Kitano H (2008). Recrystallization of water in a non-water-soluble polymer examined by Fourier transform infrared spectroscopy: poly(2-methoxyethylacrylate) with low water content. J. Phys. Chem. B.

[75] Morita S, Tanaka M, Ozaki Y (2007). Time-resolved in situ ATR-IR observations of the process of sorption of water into a poly(2-methoxyethyl acrylate) film. Langmuir.

[76] Sekine Y (2014). Dependence of structure of polymer side chain on water structure in hydrogels. Polymer.

[77] Sekine Y, Ikeda-Fukazawa T (2009). Structural changes of water in a hydrogel during dehydration. J. Chem. Phys..

[78] Kitano H, Nagaoka K, Tada S, Gemmei-Ide M (2007). Structure of water in the vicinity of amphoteric polymers as revealed by Raman spectroscopy. J. Colloid Interface Sci..

[79] Gun’ko VM (2005). Unusual properties of water at hydrophilic/hydrophobic interfaces. Adv. Colloid Interface Sci..

[80] Pissis P, Konsta AA, Ratkovic S, Todorovic S, Laudat J (1996). Temperature- and hydration-dependence of molecular mobility in seeds. J. Therm. Anal..

[81] Hackl EV, Khutoryanskiy VV, Tiguman GMB, Ermolina I (2015). Evaluation of water properties in HEA-HEMA hydrogels swollen in aqueous-PEG solutions using thermoanalytical techniques. J. Therm. Anal. Calorim..

[82] Lee HB, Jhon MS, Andrade JD (1975). Nature of water in synthetic hydrogels I. Dilatometry, specific conductivity, and differential scanning calorimetry of polyhydroxyethyl methacrylate. J. Colloid Interface Sci..

[83] Choi S, Jhon MS, Andrade JD (1977). Nature of water in synthetic hydrogels III. Dilatometry, specific conductivity, and dielectric relaxation of poly(2,3-dihydroxypropyl methacrylate). J. Colloid Interface Sci..

[84] Hirata T (2017). Dynamics of a bioinert polymer in hydrated states by dielectric relaxation spectroscopy. Phys. Chem. Chem. Phys..

[85] Naohara R, Narita K, Ikeda-Fukazawa T (2017). Change in hydrogen bonding structures of a hydrogel with dehydration. Chem. Phys. Lett..

[86] Bosio L, Johari GP, Oumezzine M, Teixeira J (1992). X-ray and neutron scattering studies of the structure of water in a hydrogel. Chem. Phys. Lett..

[87] Cavalieri F (2006). Water, solute, and segmental dynamics in polysaccharide hydrogels. Macromol. Biosci..

[88] Branca C (2002). Hydration study of PEG/water mixtures by quasi elastic light scattering, acoustic and rheological measurements. J. Phys. Chem. B.

[89] Tamai Y, Tanaka H, Nakanishi K (1996). Molecular dynamics study of polymer-water interaction in hydrogels. 2. Hydrogen bond dynamics. Macromolecules.

[90] Tamai Y, Tanaka H, Nakanishi K (1996). Molecular dynamics study of water in hydrogels. Mol. Simul..

[91] Kuo A-Y, Urata S, Koguchi R, Yamamoto K, Tanaka M (2019). Analyses of equilibrium water content and blood compatibility for Poly(2-methoxyethyl acrylate) by molecular dynamics simulation. Polymer.

