# Supplementary material for: Water as the often neglected medium at the interface between materials and biology
Source: Nat Commun. 2022 Jul 21;13:4222. doi: 10.1038/s41467-022-31889-x (PMC9304379; doi:10.1038/s41467-022-31889-x)
Supplement: Supplementary file 1 — Supplementary Information [file 41467_2022_31889_MOESM1_ESM.docx]

**Supplementary Information**

**Water as the Often Neglected Medium at the Interface between Materials and Biology**

**B. L. Dargaville^1^ and D. W. Hutmacher^1^***

1. Method details used to construct **Figure 3.**

Database: *Web of Science*

Topic: *hydrogel* swelling*

Sub-Topic: *PBS*

Years: *2000­–2021* (2022 was excluded for the sake of displaying only full-year data)

The search was limited to the following categories as deemed relevant to the topic of biomaterials and medical applications:

*Materials science biomaterials*

*Engineering biomedical*

*Pharmacology pharmacy*

*Biotechnology and applied microbiology*

*Chemistry medicinal*

*Cell tissue engineering*

*Medicine research experimental*

*Surgery*

*Dermatology*

*Orthopedics*

*Clinical neurology*

*Medicine general internal*

*Neurosciences*

*Oncology*

*Dentistry oral surgery medicine*

*Hematology*

*Neuroimaging*

*Veterinary sciences*

*Emergency medicine*

*Peripheral vascular disease*

*Cardiac cardiovascular systems*

*Critical care medicine*

*Respiratory system*

A manual check was done on each of the 127 papers returned by the refined search to ensure that PBS was mentioned in regard to the swelling medium, rather than being only used in some other context.

Search results as of 24 Feb 2022

| **Search refined by:** | **No. of results returned (since 2000)** |
| --- | --- |
| Topic: hydrogel* swelling | 18,685 |
| Limited to above categories | 3,949 |
| Refined by sub-topic: PBS | 127 |
| After manual check of content | 97 |

Yellow highlight indicates data shown in **Figure 3.**
